# Editorial: Maternal health in conflict settings: volume II

**DOI:** 10.3389/fgwh.2025.1647567

**Published:** 2025-07-24

**Authors:** Isabelle L. Lange, Ribka Amsalu, Tabassum Firoz

**Affiliations:** ^1^ Department of Infectious Disease Epidemiology and International Health, London School of Hygiene & Tropical Medicine, London, United Kingdom; ^2^ Department of Neurology, Center for Global Health and School of Medicine and Health, Technical University of Munich, Munich, Germany; ^3^ Department of Obstetrics, Gynaecology & Reproductive Sciences, University of California, San Francisco, CA, United States; ^4^ Department of Medicine and Department of Obstetrics, Gynecology and Reproductive Sciences, Yale School of Medicine and Bridgeport Hospital, Yale New Haven Health, Bridgeport, CT, United States

**Keywords:** maternal health, humanitarian health, conflict settings, women's health activism, global health research

**Editorial on the Research Topic**
Maternal health in conflict settings: volume II

Armed conflicts and humanitarian crises have profound implications for population health. Conflict often disrupts healthcare systems, increases the risk of infectious disease transmission, and exacerbates existing health inequities. The destruction of infrastructure and the displacement of populations hinder access to essential medical services, leading to heightened morbidity and mortality (1), even in spaces intended to offer respite and safety during displacement. Women are disproportionately affected in conflict settings, facing heightened risks of sexual violence, exploitation, and reproductive health challenges (2).

The breakdown of social structures and healthcare services intensifies these vulnerabilities, resulting in adverse maternal health outcomes. In 2023, countries with humanitarian response plans to an active or protracted conflict or to climate-associated crises accounted for 64% of global maternal deaths, 50% of newborn deaths, and 51% of stillbirths, according to the Mortality in Humanitarian Settings Dashboard (3). For instance, in South Darfur, Sudan, the ongoing conflict has led to a surge in maternal deaths, with sepsis and malnutrition being significant contributors (4). The devastating war in Gaza sees an estimated 17,000 pregnant and breastfeeding women needing treatment for acute malnutrition in the coming months, with serious long-term health consequences for both mothers and their infants (5), in addition to countless women who have died directly due to war and indirectly due to treatable disease conditions.

Since our first Frontiers special issue on Maternal Health in Conflict Settings (6), both the number and scale of conflicts have increased globally. In 2023, a record 59 armed conflicts were documented, the highest since data collection began in 1946 (7). This escalation has led to a substantial rise in the number of people affected by conflicts. By the end of 2023, an estimated 117.3 million individuals worldwide were forcibly displaced due to persecution, conflict, violence, human rights violations, and events seriously disturbing public order (8).

The last decade has also highlighted the weaknesses of the current humanitarian response system. The challenges of the humanitarian response system include mismatch between resources available and the humanitarian need; heavy operational cost that restricts what is available for direct assistance to beneficiaries; lack of quality data on population health, mortality or main causes of illness/death to inform public health action; heavy propaganda obscuring realities; and the top-down approach with actors in the Western Hemisphere often dictating what and who should be prioritized for humanitarian assistance. Reform is a must.

The articles in this second volume of our special issue, where we sought submissions by practitioners, policy makers, and researchers who are first-hand experiencing the harm of wars, highlight key actors and processes that play fundamental roles in maternal and reproductive health in humanitarian crisis zones. They urge us to think broadly about who is affected and which strategies can improve health, systems, and care in these settings.

The disconnect between need vs. resources committed for maternal and newborn health in humanitarian settings could not be starker when one reads Adler et al.'s article, *Unpacking global stakeholder perspectives on factors that influence the prioritization of MNH in humanitarian settings on the global health agenda.* Funding for maternal and newborn health has declined in the post-MDG era, and there is limited political will, continued fragmentation between the humanitarian and development community; inability or unwillingness to lead from behind by some donors, UN and international non-profit actors; and preference for quick wins.

Hafez et al., in a compelling narrative, take readers to the Nuba Mountains, and how communal self-care remains the bedrock for women going through pregnancy, childbirth and the postnatal period in the absence or limited availability of formal healthcare and the unpredictable cycle of war, displacement and trauma [*Self-care for maternal and reproductive health in conflict settings: qualitative case study in Nuba Mountains, Sudan*]. The article gives readers a glimpse of day-to-day survival strategies of women and how small or insignificant humanitarian assistance is available for women in the Nuba Mountains. The authors urge health responses to build on existing communal self-care systems.

It is rare to have population-based data on the magnitude of gender-based violence (GBV) in conflict settings. Asefa et al. do that in their article. *The magnitude of gender-based violence, health consequences, and associated factors among women living in post-war woredas of North Shewa zone, Amhara, Ethiopia, 2022*. A lot has been written on how GBV is used as a weapon of war and how current preventative systems and care for survivors are inefficient and unfit for purpose. The authors found gaps in the legal and health response and draw attention to the field reality that when social systems are disrupted during war, the perpetrators are not restricted to state and non-state armed actors. The article is similar with Hafez's research in the Nuba Mountains in that humanitarian actors are invisible in the humanitarian response to the conflict in the Amhara region of Ethiopia.

Two qualitative studies from Somalia and Yemen underscore the role of midwives in conflict settings and the impact of conflict on their career choice and how they deliver care. In the study by Abdullahi et al. [*Experiences of midwifery students and graduates in Somalia: evidence from qualitative data*], midwifery students and recent graduates cited several barriers to entering the workforce. Participants identified that conflict impacts their ability to regularly attend school and expressed concern about accepting jobs in remote areas due to threats to their safety. While they mostly felt prepared to practice as midwives, knowledge gaps included abortion care, neonatal resuscitation, and usage of basic ultrasound.

Similarly, Al Zumair et al. [*Midwives’ experiences working with women and girls surviving violence in Yemen: a qualitative study*]. Found that conflict led to multi-level barriers to midwifery care of Yemeni women facing GBV including lack of training, treatment guidelines and supportive services as well the stigma of gender-based violence. The study which explored community midwives’ knowledge, training and experience working with women and girls who experience interpersonal violence in Yemen found that midwives play a critical role in responding to gender-based violence in by providing psychological support, violence-related health care, and referrals to medical and protection services.

These studies underscore that communities at the epicenter of humanitarian crises are the first responders. Investing in training for local responders and supporting academic and programmatic institutions in the Global South could help address a key limitation of the current humanitarian system: its inability to meet rising needs efficiently and cost-effectively.

Despite all the challenges described above, the systematic review by Kasonia et al. [*Pregnancy and neonatal outcomes in Eastern Democratic Republic of the Congo: a systematic review*] gives reason for optimism. This comprehensive overview of maternal and neonatal outcomes over a 20-year period in the Eastern Democratic Republic of the Congo (DRC) found that while the data quality was variable, the rates of maternal and neonatal complications in the Eastern DRC are comparable with those observed in other countries in the region that are not affected by armed conflict. The study calls for exploration of the resilience of the DRC health system, which could be valuable for other health systems to learn from.

Core themes from the first volume – the need for reliable data, the importance of comprehensive maternal health care provision, and the recognition of experiences of conflict settings to inform implementation strategies (9) – are threaded through the publications in this volume. The analyses presented here reinforce calls to move beyond standard health measures (10), highlighting the many ways in which the social, psychological, and interpersonal facets of humanitarian crises shape the lived experiences of those affected, particularly girls and women. As a recent report suggests, while women are among the most affected by conflict, they also play a crucial role in peacebuilding processes (2). This underscores the need for women's health to be recognized not only as a health issue but as a fundamental element of conflict resolution and recovery efforts. Women led for-profit and non-profit organizations are needed in conflict and crisis prone countries to fundamentally reform the sector.

Addressing long-term development needs in post-conflict and protracted crisis settings is essential. These contexts require different skills and approaches than acute emergency responses, highlighting the need for tailored training and more implementation research to build evidence on effective, scalable interventions. Research and innovation must be informed and shaped by the needs and priorities of end users – including communities, governments, and non-governmental organizations – if they are to truly transform the humanitarian sector. One such area that offers promising tools is artificial intelligence, including for rapid assessment (e.g., estimating population size, mapping access to water, hospitals and other services, identifying disease risk); improving quality of care through predictive analytics; telemedicine and enabling real-time data analysis for timely action.

We hope for this special issue to contribute to the scientific evidence base and support advocacy efforts to update global health policies, positioning women's health as a core component of humanitarian relief and conflict response. Recognizing the critical role of women's health in peacebuilding and reconstruction agendas is essential for fostering resilience and long-term recovery in conflict-affected communities.

## References

[1] Murray CJ King G Lopez AD Tomijima N Krug EG. Armed conflict as a public health problem. Br Med J. (2002) 324(7333):346–9. 10.1136/bmj.324.7333.346 11834565 PMC1122272

[2] Chan W. War’s disproportionate impacts on women. Carnegie Reporter. Volume 15, Number 1. New York, NY: Carnegie Corporation of New York (2024).

[3] AlignMNH, Interagency Working Group on Reproductive Health in Crises, Jhpiego. Maternal and Neonatal Mortality in Humanitarian Settings Dashboard. Baltimore, Maryland: MNH Targets, Measurement, & Data (2022).

[4] AP News. Sepsis and Malnutrition Stalk the new Mothers and Babies of South Darfur. New York City, NY: Associated Press (2024). (Accessed February 10, 2024).

[5] World Health Organization (WHO). People in Gaza starving, sick and dying as aid blockade continues. Available online at: https://www.who.int/news/item/12-05-2025-people-in-gaza-starving-sick-and-dying-as-aid-blockade-continues (Accessed May 21, 2025)

[6] Firoz T Tappis HL Lange IL Amsalu R. Maternal health in conflict settings. Front Glob Womens Health. (2022) 3:807257. 10.3389/fgwh.2022.807257 35368994 PMC8964459

[7] Uppsala University. New Data Indicate Record Number of Armed Conflicts in the World June 3rd. Isle of Man: Science X Network (2024). https://phys.org/

[8] United Nations High Commissioner for Refugees (UNHCR). Global Trends: Forced Displacement in 2023. Copenhagen, Denmark: United Nations High Commissioner for Refugees (2024).

[9] Amsalu R Firoz T Lange IL Tappis H. Maternal health in conflict settings. Front Glob Womens Health. (2022) 3:807257. 10.3389/fgwh.2022.807257 35368994 PMC8964459

[10] Garry S Checchi F. Armed conflict and public health: into the 21st century. J Public Health. (2020) 42(3):e287–98. 10.1093/pubmed/fdz095 31822891

